# Hyaluronic Acid Derivative Molecular Weight-Dependent Synthesis and Antimicrobial Effect of Hybrid Silver Nanoparticles

**DOI:** 10.3390/ijms222413428

**Published:** 2021-12-14

**Authors:** Guillem Ferreres, Sílvia Pérez-Rafael, Juan Torrent-Burgués, Tzanko Tzanov

**Affiliations:** Grup de Biotecnologia Molecular i Industrial, Department of Chemical Engineering, Universitat Politècnica de Catalunya, Rambla Sant Nebridi 22, 08222 Terrassa, Spain; guillem.ferreres@upc.edu (G.F.); silvia.perez.rafael@upc.edu (S.P.-R.); juan.torrent@upc.edu (J.T.-B.)

**Keywords:** hyaluronic acid, adipic acid dihydrazide, silver nanoparticles, polymer molecular weight, antimicrobial

## Abstract

Silver nanoparticles (Ag NPs) appeared as promising antimicrobial candidates to face the development of antibiotic resistance. Although reported as toxic towards mammalian cells, their combination with biomolecules have shown reduced toxicity, while maintaining the antimicrobial function. Herein, hyaluronic acid (HA) with low (40 kDa), medium (200 and 600 kDa) and high (2 MDa) molecular weight (Mw) was modified with adipic acid dihydrazide (ADH) and used as reducing and capping agents to synthesise antimicrobial hybrid Ag NPs. The Mw of the polymer played a crucial role in the morphology, size and antibacterial activity of the Ag NPs. The 600 and 200 kDa HA-ADH-Ag NPs were able to reduce the *Escherichia coli* and *Staphylococcus aureus* concentration by more than 3 logs, while the 40 kDa NPs reached ~2 logs reduction. The 2 MDa HA-ADH failed to form homogenous NPs with strong bactericidal activity. A mechanistic study of the interaction with a model bacterial membrane using Langmuir isotherms confirmed the greater interaction between bacteria and higher Mw polymers and the effect of the NP’s morphology. The nanocomposites low toxicity to human skin cells was demonstrated in vitro, showing more than 90% cell viability after incubation with the NPs.

## 1. Introduction

Inefficacy of antibiotics due to the advent of multidrug resistance of pathogens has become a global health emergency, causing complicated infections and even death. Conventional antibiotics have very specific mechanisms of action, which enable the development of resistance associated to single mutations sufficient to counteract the effect of the drug [1,2]. Conversely, metal (silver [3] and copper [4]) and metal oxide (zinc oxide [5]) nanoparticles (NPs), possess multiple antimicrobial mechanisms, comprising membrane disruption, ion release, ROS formation, inactivation of bacterial enzymes and metabolic pathways and damage of pathogen’s DNA [6]. Simultaneous and unspecific mechanisms broaden the spectrum of antibacterial activity and hamper the resistance acquisition due to multiple mutations required to overcome the antimicrobial action. Compared to their bulk forms, NPs possess larger surface area to volume ratio allowing stronger interaction with bacterial cell structures, resulting in higher antimicrobial efficiency. Despite the potential of these materials as antimicrobial agents, they have been described as toxic towards mammalian cells [7]. To avoid their intrinsic toxicity these nano-antimicrobials can be combined with biomolecules, for both enhanced biocompatibility [8] and antimicrobial efficacy [9].

Silver is still by far the most extensively used antimicrobial metal for biomedical applications. Biopolymers, such as polysaccharides, have been increasingly employed as natural alternatives to traditional chemical reducing and capping agents for Ag NPs synthesis, providing multiple benefits as: (i) NPs’ stabilisers prolonging their shell-life, (ii) protective agents that decrease the NP toxicity to human cells, (iii) enhancers of the antimicrobial properties of Ag NP and (iv) providers of functional groups allowing immobilisation on solid substrates or further surface functionalisation. Positively charged biopolymers, such as chitosan and aminocellulose, are well-known antimicrobials agents, which interact with bacterial membranes, producing changes in their permeability, homeostasis failure and membrane hydrolysis, ultimately resulting in cell death [10]. Conversely, biopolymers without antimicrobial activity, e.g., hyaluronic acid, enhance the biocompatibility of toxic antimicrobial agents towards mammalian cells [11]. Moreover, their functional groups allow for further modifications to provide additional bioactivities. In the synthesis of metal NPs, these biomacromolecules may serve as reducing agents of the corresponding metal salt to generate stable NPs dispersion [12,13,14].

Herein, a hydrazide-modified hyaluronic acid (HA) was synthesised and used for production of antimicrobial silver-enabled nanocomposites. HA is a linear glycosaminoglycan formed by alternating units of β-1,4-linked D-glucuronic acid and β-1-3-linked N-acetyl-D-glucosamine. It is the main structural component of the extracellular matrix involved in signalling processes, inflammation, wound repair and morphogenesis, among others. Furthermore, the biological function of HA is determined by its molecular weight (Mw). For instance, low Mw stimulates inflammation, while higher chain length induce anti-inflammatory response [15]. On the other hand, adipic acid dihydrazide (ADH) is a low toxicity ligand that have been described to form complexes with metal ions such as Ag^+^ or Cu^2+^ [16,17].

This study attempts to achieve green synthesis of stable and concentrated dispersions of antimicrobial HA-derivative capped Ag NPs using biopolymer-assisted Ag^+^ reduction into Ag NPs. HA was modified with adipic acid dihydrazide (HA-ADH) in order to promote the interaction between silver ions and the biopolymer, and thus enhancing its potential as a reducing and capping agent in the synthesis of Ag NPs. Since little is known on how the polymer Mw influences the NPs synthesis, the size/distribution and antibacterial efficacy of HA-ADH-Ag NPs were studied using HA-derivatives with increasing molecular weight (40, 200, 600 kDa and 2 MDa).

## 2. Results and Discussion

### 2.1. Amination of HA

**Scheme 1:**
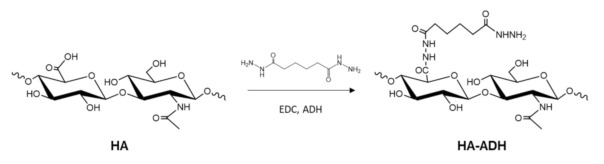
Hyaluronic acid modification with adipic acid dihydrazide.

**Figure 1:**
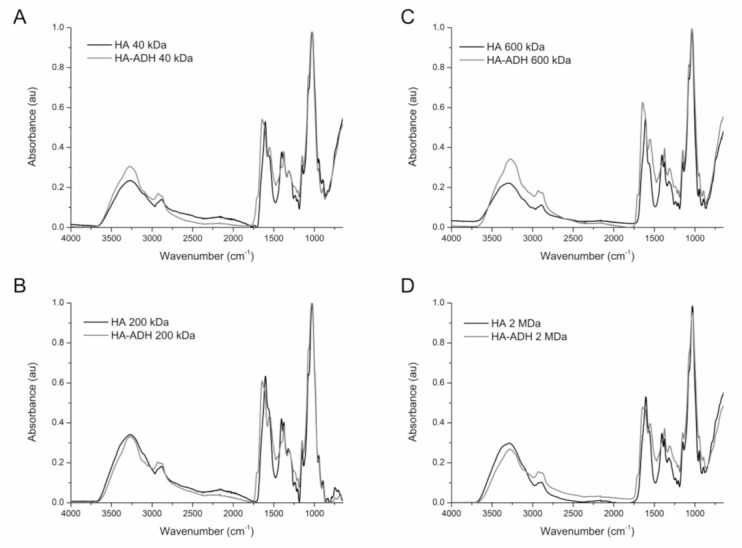
FTIR spectra of pristine and ADH-modified hyaluronic acid: (**A**) 40 kDa, (**B**) 200 kDa, (**C**) 600 kDa and (**D**) 2 MDa.

Biopolymers have been widely used to synthesise nanocomposite materials, yielding hybrid structures with a broad range of functionalities and superior performance compared to their individual constituents. In the synthesis of Ag NPs, the biopolymers are involved in both silver ion reduction and capping, thus overcoming the major drawbacks of traditional chemical synthetic methods, such as the use of chemical reducers or high temperature. However, pristine HA is not able to produce metal NPs without these conditions [18,19]. In the current work, the modification of HA with ADH moieties was expected to promote the metal coordination-driven interaction between the silver ions and HA, and enhance the hybrid HA-ADH-Ag NPs formation. The conjugation reaction was performed using 1-ethyl-3-(3-dimethylaminopropyl)carbodiimide (EDC), which activated the carboxylic groups in HA for reacting with the amino groups of ADH (Scheme 1) [20]. In the FTIR spectrum of HA-ADH, compared to unmodified HA, the presence of the signal at 1281 cm^−1^ corresponded to the stretching vibration of C-N bonds from the ADH functional groups. The new amide I (1650 cm^−1^) and amide II (1535 cm^−1^) peaks confirmed the presence of the amide groups of ADH [21]. The signal at 3000 cm^−1^ in the pristine HA samples split into two signals in the modified samples due to the stretching vibration of the C-H bonds of ADH. Finally, the subtle change in the shape of the signal at 3300 cm^−1^ could correlate with N-H vibration [16]. These changes were observed in the spectra of the four modified HA samples when compared to their unmodified counterparts, indicating that the ADH-grafting reaction was successful regardless the Mw of HA (Figure 1). The degree of HA amination (~60%), quantified by the trinitrobenzene sulfonic acid (TNBSA) assay, was similar for the low and medium Mw HA samples. On the other hand, the 2 MDa showed slightly lower modification (~45%) (Figure S1).

### 2.2. HA-ADH-Ag NPs Characterisation

**Figure 2:**
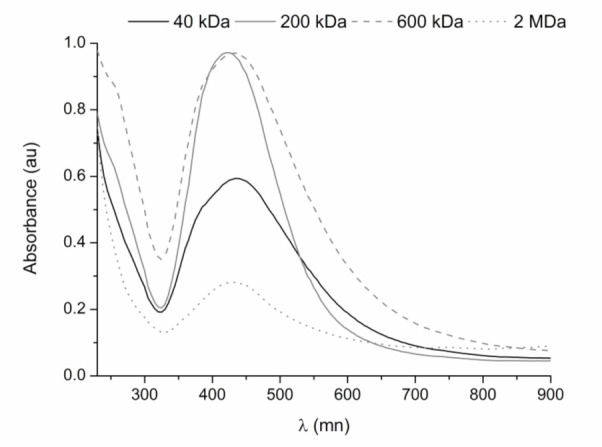
UV-vis spectra of HA-ADH-Ag NPs dispersions synthesised with different Mw HA-ADH derivatives.

**Figure 3:**
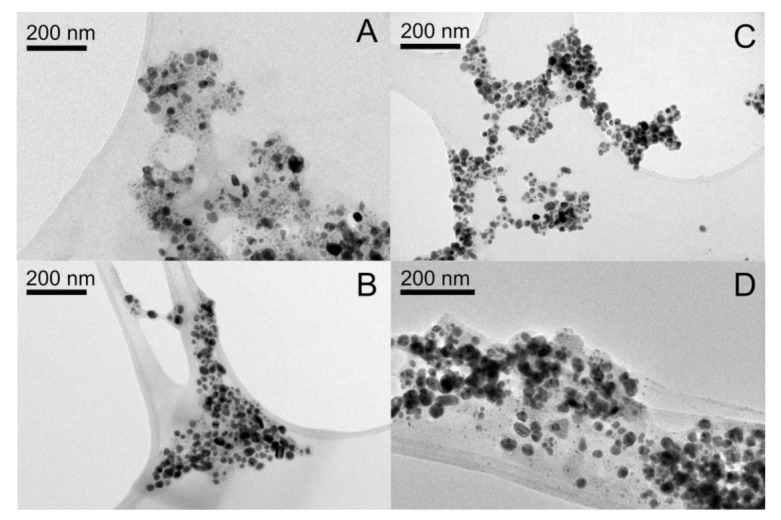
TEM images of HA-ADH-Ag NPs synthesised with (**A**) 40 kDa, (**B**) 200 kDa, (**C**) 600 kDa and (**D**) 2 MDa.

The synthesis of stable and concentrated dispersion of HA-ADH-Ag NPs were achieved upon mechanical stirring of four different Mw HA-ADH (40, 200, 600 kDa and 2 MDa) solutions with silver nitrate at room temperature for 24 h. In this study, the different polymer molecular weights were used to investigate the effect of the chain length on the NPs size/distribution and their antimicrobial performance. After centrifugation and separation process to remove the unreacted precursors, the NPs dispersions presented turbidity and brownish colour [22]. The Ag NP formation was confirmed by UV-vis spectrophotometry. Unlike the unmodified HA (Figure S2), all the samples prepared with HA-ADH showed absorbance spectra with the characteristic peak of nano-silver at 420 nm (Figure 2) [23]. Besides this, the spectra corresponding to medium Mw polymer (200 and 600 kDa) showed narrower and more intense absorbance peak than the low and high molecular weight counterparts [24]. TEM analysis demonstrated that the Mw of the biopolymer determined the particle size and polydispersity. HA-ADH with 200 and 600 kDa produced uniform spherical HA-ADH-Ag NPs of approximately 18 nm, whereas the Ag NPs prepared with low Mw (40 kDa) displayed irregular form and heterogeneous size distribution (Figure 3). The nanoparticulate conjugates obtained with 2 MDa HA-ADH were larger (around ~20–40 nm), with irregular forms (images showed spherical, amorphous, oval and triangular morphologies) and higher polydispersity (Figure 3D and Figure S3). The size and polydispersity of hybrid NPs are typically controlled by varying the temperature, reaction time and ratio of the reagents used. Herein, the results indicated that the size and polydispersity of the NPs could be tuned simply by varying the biopolymer Mw. The presence of the hydrophilic biopolymer on the surface of the NPs provided a molecular barrier that acts as a stabiliser for the nanocomposites through steric repulsions, preventing them from agglomeration. As reported, such effect is enhanced by the length of the polymer chain [25,26,27]. Similarly, higher Mw polyvinyl alcohol and chitosan used as silver reducing agents yielded smaller and more stable Ag NPs than their lower Mw counterparts [28,29]. At the same mass concentration, the HA-ADH solution of low Mw contains more macromolecules than the corresponding solutions of higher Mw biopolymer. Higher HA-ADH molecular concentration might boost the HA-ADH/silver nanostructures aggregation, resulting in larger nanoparticles. It is reported that high Mw HA solutions present pseudo-gel-like behaviour due to polymer chain entanglement [30]. The viscosity of 2 MDa solutions drastically increased compared to the 40, 200 and 600 kDa samples, which may hinder the diffusion of the metal ions through the polymeric matrix, favouring the formation of bigger metal-polymer clusters with irregular shape.

### 2.3. Mechanism of HA-ADH-Ag NPs Formation

**Figure 4:**
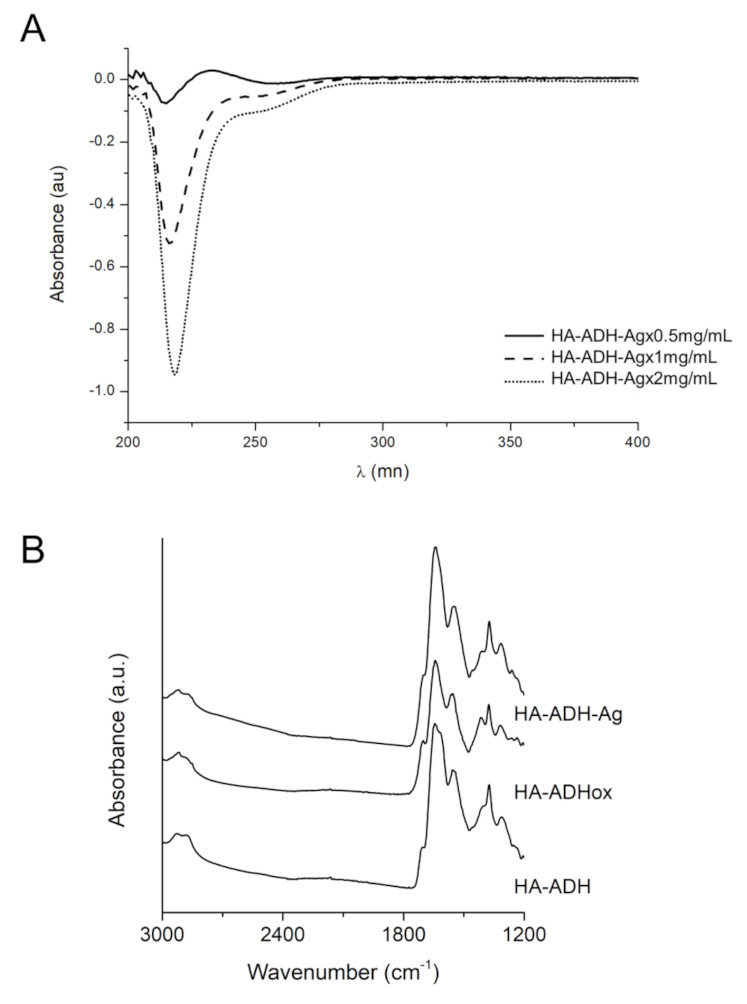
(**A**) UV-vis spectrum of the HA-ADH subtracted to the spectra of HA-ADH/Ag^+^ mixtures. (**B**) FTIR spectra of 200 kDa HA-ADH, HA-ADH-Ag NPs and HA-ADH oxidised with sodium periodate (HA-ADHox).

Metal ion-biopolymer interaction has been identified as a key factor in the hybrid metal-organic NPs synthesis. Although HA could interact with Ag^+^ through glucosidic groups, the unmodified polymer failed to form NPs. Therefore, the capacity of hydrazides to form complexes with metal ions was explored to enhance the interaction of the polymer with Ag^+^ during the NPs formation [31,32]. In order to assess the interaction between the silver ions and HA-ADH, the UV-vis spectra of HA-ADH/Ag^+^ mixtures were recorded. The addition of silver salt increased the signal at ~220 nm and shifted it towards longer wavelength, which correlate with a coordination reaction between HA-ADH and Ag^+^ (Figure 4A and Figure S4). This interaction makes available and exposes Ag^+^ to reduction by the -OH groups in HA, yielding HA-ADH-Ag NPs. The FTIR analysis of the NPs revealed the increase of the following signals in HA-ADH spectrum: at 2859 and 1297 cm^−1^ corresponding to C-H stretching and bending vibration from aldehydes, at 1693 cm^−1^ assigned to C=O stretching vibration, and at 1420 cm^−1^ corresponding to the increase of carboxylic groups. These changes were observed in the spectra of 200, 40 and 600 kDa (Figure 4B and Figure S5). The same signals appear in HA-ADH treated with sodium periodate (Figure 4B) due to the formation of carbonyl groups by oxidation of C2 and C3 and opening of the glucuronic acid ring of HA [33]. Thus, we hypothesised that the oxidation of HA-ADH during the Ag NPs formation follows a similar pattern.

### 2.4. Antibacterial Activity of HA-ADH-Ag NPs

**Figure 5:**
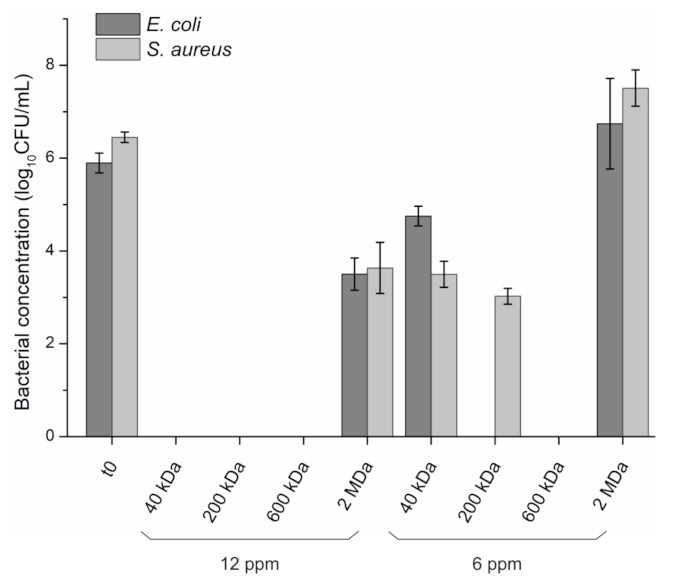
Antimicrobial activity of HA-ADH-Ag NPs synthesised using 40, 200, 600 kDa and 2 MDa HA-ADH towards *E. coli* and *S. aureus.* t0 indicates the initial bacterial concentration of the test.

The antibacterial efficacy of HA-ADH-Ag NPs were assessed towards two medically relevant bacterial pathogens: the Gram-negative *E. coli* and the Gram-positive *S. aureus*. Biocidal activity of the nanocomposites was expected due to the generation of ROS and release of Ag^+^ [34,35]. In order to compare the antibacterial activity of HA-ADH NPs obtained from HA-ADH of different Mw (40, 200, 600 kDa and 2 MDa), each NPs solution was prepared at the same silver concentration. Interestingly, significant differences in the antibacterial performance of the four sets of NPs were observed. At a total silver concentration of 12 ppm, both bacteria were completely eradicated by 40, 200 and 600 kDa HA-ADH-Ag NPs, however, the 2 MDa NPs were only able to reduce ~2.5 logs of the bacterial concentration. The NPs of 600 kDa HA-ADH, containing 6 ppm silver, efficiently eradicated both bacteria, while those prepared with 200 kDa HA-ADH at the same silver concentration caused full killing of *E. coli* and 3 logs reduction of *S. aureus* concentration. On the other hand, the HA-ADH-Ag NPs made of 40 kDa HA-ADH at 6 ppm of silver presented lower activity, reducing less than 3 logs the bacterial concentration. Finally, the ones prepared with 2 MDa HA-ADH did not show bactericidal activity at this concentration towards any of the tested bacterial species (Figure 5). Although HA-ADH in bulk form did not display antimicrobial activity, different Mw HA derivatives unexpectedly modulated the antimicrobial activity of the nanocomposite. The impact of the chain length on the antimicrobial activity has been previously described for other biomolecules, such as chitooligosaccharides [36]. On the other hand, the size and shape play a crucial role in the NPs activity. It has been reported that small changes in the size of the particles can affect their antimicrobial potential, generally improving with smaller particle sizes [37,38]. Accordingly, the irregular morphology of the 40 kDa and 2 MDa NPs may reduce their interaction with the bacterial membranes, and therefore, their antimicrobial efficacies [39,40].

### 2.5. HA-ADH-Ag NPs Interaction with a Bacterial Model Membrane

**Figure 6:**
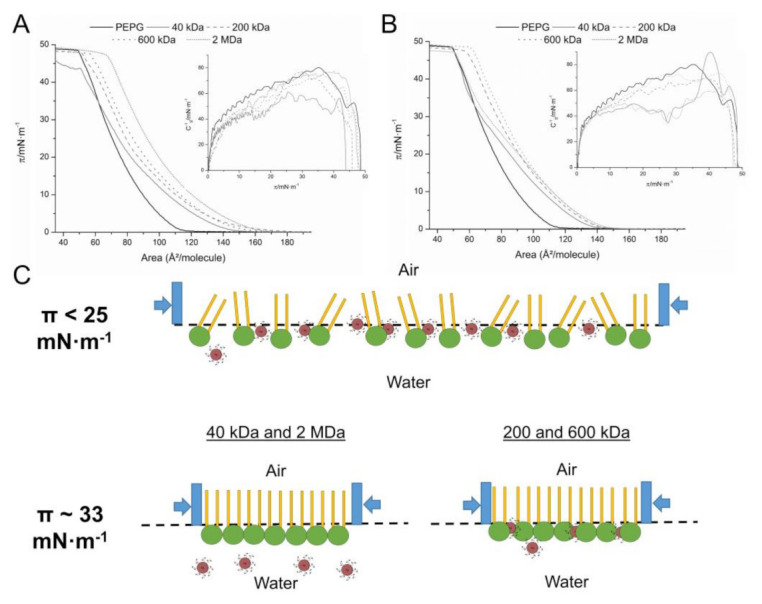
π-A isotherms of a PE:PG 8:2 mix monolayers in PBS subphase (solid black), or with HA-ADH in bulk form (**A**) or in nano-form (**B**) of 40 kDa (grey solid), 200 kDa (grey dash), 600 kDa (grey dot) and 2 MDa (grey short dot). Insets: the inverse of the compressibility modulus. (**C**) Cartoon illustrating the interactions of the HA-ADH-Ag NPs with the model membrane.

In order to elucidate the effect of the Mw on the antimicrobial performance of the nanocomposites, the interactions between the HA derivatives and a mimetic Gram-negative bacterial membrane model were studied using Langmuir isotherms. The monolayer was formed using the two main phospholipids of the *E. coli* membrane-phosphatidylethanolamine (PE): phosphatidylglycerol (PG) [41]. Similar to previous studies, the PE:PG π-A isotherm presented a monotonic increase until the collapse pressure (CP) at π ~48 mN·m^−1^ [42]. As expected, unmodified HA did not produce significant changes in the compression isotherm for *E. coli* model membrane (Figure S6). In contrast, at low lateral pressures (π < 25 mN·m^−1^), the addition of HA-ADH and HA-ADH NPs shifted the control PE:PG isotherm (Figure 6A,B, black line) towards higher values of area per molecule. Analogous behaviour has been reported for membrane disturbing biopolymers such as chitosan [43]. The increase of the area observed may be due either to intercalation of the polymer/NPs between the phospholipids chains, or to electrostatic interactions [44]. At π > 25 mN·m^−1^, the 40 kDa HA-ADH in bulk and nanoform behave similar to the PE:PG, but presented slightly lower CP. In the case of 200 and 600 kDa HA-ADH in both forms, the initial shift was maintained, increasing the area per molecule, however, the CP was the same as in the PE:PG isotherm. Finally, the highest Mw HA-ADH (2 MDa) promoted the interaction and shifted the position of the isotherm towards higher area per molecule values (Figure 6A). On the other hand, 2 MDa capped Ag NPs did not interact with a detectable affinity with *E. coli* model membrane (Figure 6B).

The effect of the NPs and biopolymers on the lipid monolayer was measured comparing the area of the isotherms at 33 mN·m^−1^, corresponding to the physiological membrane pressure [45]. At this pressure, the PE:PG monolayers with PBS, 40 kDa HA-ADH and 40 kDa HA-ADH-Ag NPs presented an area of ~63 Å^2^/molecule, indicating that at this pressure, the 40 kDa samples were not interacting with the model membrane. On the other hand, the 200 kDa and the 600 kDa HA-ADH showed areas of 71 and 74 Å^2^/molecule in bulk form, and 73 and 78 Å^2^/molecule in nanoform, respectively. Finally, 2 MDA HA-ADH presented an area of 82.4 Å^2^/molecule and 65.3 Å^2^/molecule in NPs form. Despite not having antibacterial activity, the HA-ADH polymer displayed almost a linear area increment (from 63–82.4 Å^2^/molecule) with higher Mw, which may be correlated with stronger interactions with the bacterial membrane (Figure S7). From low to medium Mw polymer NPs, the interaction with the PE:PG monolayer is clearly enhanced by HA-ADH polymer chain length. In agreement with the antibacterial properties, polymer-capped Ag NPs with higher Mw (600 kDa) presented stronger activity than NPs with similar sizes and morphology (40 and 200 kDa). The drastic interaction loss of the 2 MDa NPs (65.3 Å^2^/molecule) may be due to the higher size, polydispersity and heterogeneous shape of the NPs. The Langmuir study results showed the contribution of both factors: (i) biopolymer Mw and (ii) NPs size, morphology and polydispersity, in the interaction of the nanocomposite with the bacterial surface.

In the case of the inverse of the compressibility module curves, although the PE:PG monolayer is maintained in a fluid or liquid state, the NPs induced a decrease in the Cs^−1^ values (Figure 6A,B), indicating an increase of the membrane fluidity [46]. As this effect could not be correlated with the antimicrobial-Mw tendency observed in the antimicrobial assay, we assumed that it may not have an important contribution in the antimicrobial mechanism of the NPs.

### 2.6. Biocompatibility of the HA-ADH-Ag NPs

**Figure 7:**
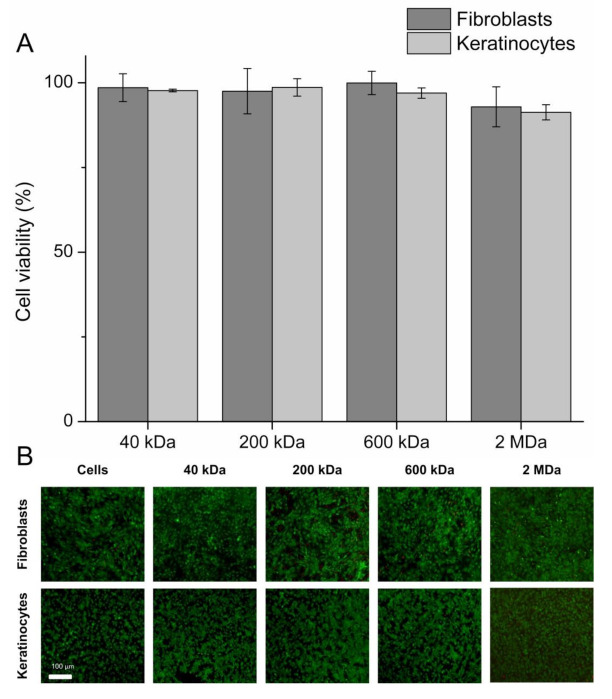
Cytotoxicity of HA-ADH-Ag NPs synthesised using 40, 200, 600 kDa and 2 MDa HA-ADH at 6 ppm of silver content. (**A**) AlamarBlue assay for assessment of the cellular metabolic activity of fibroblast (BJ5tα) and keratinocytes (HaCat) after 24 h incubation with HA-ADH-Ag NPs. (**B**) Fluorescence microscopy images of fibroblasts (BJ5tα) and keratinocytes (HaCat) after 24 h incubation with HA-ADH-Ag NPs. The green (live cells) and red (dead cells) fluorescence images are overlaid in one picture for better comparison.

The unspecific and multiple antimicrobial mechanism of Ag NPs has been reported to cause toxicity towards mammalian cells [47]. Although the HA used in this work has been modified, it did not show any toxicity against human skin cells at a concentration of 0.9% (*w*/*v*) (Figure S8) [48]. The NPs synthesised with different Mw HA-ADH were tested with fibroblasts (BJ5tα) and keratinocytes (HaCat) at antimicrobial concentration of 6 ppm silver for 24 h. After one day of direct incubation and a further recovery time of 24 h, none of the four HA-ADH-Ag NPs was toxic towards the skin cells, which showed viability higher than 90% (Figure 7A). The fluorescence microscopy images confirmed that the HA-ADH-Ag NPs did not affect the morphology of the human skin cells (Figure 7B).

## 3. Materials and Methods

### 3.1. Reagents and Enzymes

Pharmaceutical grade hyaluronic acid (HA) sodium salt from *Streptococcus Equi* (MW = 40, 200, 600 kDa and 2 MDa) was obtained from Lehvoss Iberia (Barcelona, Spain). Silver nitrate, Muller-Hinton Broth (MHB), Coliform ChromoSelect agar, Braid-Parker agar, Dulbecco’s modified Eagle’s medium—high glucose (DMEM) were supplied by Sigma-Aldrich (Madrid, Spain). Invitrogen Life Technologies Corporation (Sant Cugat del Vallès, Spain) provided the alamarBlue cell viability reagent. Adipic acid dihydrazide (ADH), hydrochloric acid, nitric acid, 1-ethyl-3-(3-dimethylaminopropyl)carbodiimide (EDC) and 2,4,6-trinitrobenzene sulfonic acid (TNBSA) were supplied by Thermo Fischer Scientific (Sant Cugat del Vallès, Spain). Sodium dodecyl sulfate was purchased from Fisher Bioreagent (Madrid, Spain). Bacterial strains *Staphylococcus aureus* (ATCC 25923) and *Escherichia coli* (ATCC 25922), and human fibroblast (ATCC-CRL-4001, BJ-5ta) and keratinocyte (HaCaT cell line) cells were obtained from the American Type Culture Collection (ATCC LGC Standards, Barcelona, Spain).

### 3.2. Hyaluronic Acid Modification

Four HA Mws (40, 200, 600 kDa and 2 MDa) were modified with adipic acid dihydrazide as previously described [49]. Briefly, each HA was dissolved in MilliQ water at a final concentration of 2.5 mg/mL. When the HA was completely dissolved, adipic acid dihydrazide (ADH) was added in 1:40 (HA:ADH) molar ratio. After 30 min of stirring at room temperature, the pH was adjusted to 4.8 with hydrochloric acid. Subsequently, EDC was added in a molar ratio of 1:4 (HA:EDC). The reaction was performed for 2 h, adjusting the pH every 10 min to 4.8. Then, the HA was dialysed four times with distilled water to remove the unreacted compounds, the first one in 20% (*v*/*v*) of ethanol, and subsequently freeze it overnight at −80 °C. Thereafter, the HA was freeze-dried for 3 days. The HA modification was assessed through FTIR, performing 64 scans over 4000–650 cm^−1^ range with PerkinElmer Spectrum 100 (PerkinElmer, Boston, MA, USA). In order to assess the modification degree of the polymer, the TNBSA assay was performed. Briefly, 500 μL of sample and standard solutions were reacted with 250 μL TNBSA solution 0.01% (*w*/*v*) prepared in 0.1 M sodium bicarbonate buffer pH 8.5. After 2 h of incubation at 37 °C, 250 μL of 10% SDS and 125 μL of 1 M HCl were added to each sample to stop the reaction. Finally, 200 µL of all reaction were transferred to a 96-well plate and the absorbance of the solutions was measured at 335 nm.

### 3.3. Synthesis of NPs

Modified-HA of each Mw was resuspended in water to obtain 1.5% (*w*/*v*) solution. After complete dissolution, 2 mg/mL silver nitrate was added in a volume ratio of 1.5:1 (HA: silver nitrate) and stirred at 300 rpm for 24 h at room temperature. Since the 2 MDa polymer was not dissolved at a concentration of 1.5% (*w*/*v*), the HA-ADH solution was prepared at the final concentration of synthesis reaction 0.9% (*w*/*v*) and the silver salt was added in solid and subsequently the mix was strongly vortexed in order to dissolve the salt. After that, the particles were separated by centrifugation at 18,000× *g* for 40 min. The particles were washed twice with miliQ water, and finally resuspended in miliQ water.

### 3.4. Characterisation of the NPs

The study of NPs size and morphology was performed using a JEOL JEM-2100 LaB6 transmission electron microscope (TEM) operating at an accelerating voltage of 200 kV with holey carbon grid.

The NPs were analysed spectrophotometrically over the 230–930 nm range, using a microplate reader Infinite M200 (Tecan, Grödig, Austria) to identify the characteristic absorption peak of nano-silver at 420 nm. The silver content of the HA-ADH-Ag NPs was assessed using ICP. The sample preparation consisted in dissolving the NPs with 20% (*v*/*v*) nitric acid at 100 °C for 60 min. Thereafter, the volume was completed to 5 mL with ultrapure water to reach a final concentration of 2% (*v*/*v*) nitric acid. Then the samples were filtered to remove any remaining solid and the measurement was performed using an ICP-MS 7800 (Agilent Technologies, Madrid, Spain) calibrated by internal standard with ^45^Rh and a standard curve of ^107^Ag.

### 3.5. Mechanism of NPs Formation

HA-ADH and ADH with 0.5, 1 and 2 mg/mL silver nitrate were analysed spectrophotometrically using a Varian Cary 100 Bio spectrophotometer (Varian, Belrose, Australia) over a wavelength range of 200 to 400 nm in 1 cm quartz cuvettes. The HA-ADH-Ag NPs were lyophilised and compared to the HA-ADH by FTIR, performing 64 scans over the 4000–650 cm^−1^ range with PerkinElmer Spectrum 100 (PerkinElmer, Boston, MA, USA).

### 3.6. Antibacterial Activity of HA-ADH-Ag NPs

The antibacterial activity of the HA-ADH-Ag NPs was assessed towards *Escherichia coli* and *Staphylococcus aureus*. The NPs solutions at different concentrations were mixed with 50 µL bacterial inoculum at optical density (OD) at λ = 600 nm OD600 = 0.01 (~10^5^–10^6^ colony forming units (CFU) mL^−1^) in a 96-well polystyrene plates. The samples were incubated for 24 h at 37 °C with shaking at 230 rpm. A microplate reader was used to assess the bacterial growth in presence of the NPs measuring at 600 nm (OD600). Samples without turbidity (OD600 ~ 0) were considered antibacterial. Afterwards, the number of survived CFU was assessed by plating 10 µL of the suspensions onto coliform Agar for *E. coli* and Braid Parker Agar for *S. aureus*. The agar plates were incubated for 24 h at 37 °C and counted. All results are reported as mean value ± standard deviation (*n* = 3).

### 3.7. Langmuir Experiments

The capacity of the modified HA and NPs to interact with a model Gram-negative membrane was studied under biomimetic conditions by means of the Langmuir technique. Monolayers of phosphatidylethanolamine (PE): phosphatidylglycerol (PG) in a ratio of 8:2, using subphases with PBS, PBS + HA-ADH and PBS + NPs, were formed in a Langmuir trough (KSV NIMA Langmuir–Blodgett Deposition Troughs, model KN2002, Finland) equipped with two mobile barriers mounted on an antivibration table, housed in an insulation box at 23 ± 1 °C. The surface pressure (π) was measured using a Wilhelmy plate connected to the trough. The system was cleaned twice with chloroform and several times with water until the pristine subphase confirmed the cleanliness of the system. The experiments were performed with a barrier closing rate of 15 cm^2^ · min^−1^. The subphases were prepared using 100 mM PBS at pH 7.4 and 0.05% (*w*/*v*) of HA-ADH, and the NPs samples were normalised regarding the HA-ADH content determined by TNBSA. After the establishment of the subphases, 30 µL of the phospholipid mixture at a concentration of 0.5 mg/mL dissolved in chloroform was gently added on the surface of the subphase. The recording of the surface pressure-area per molecule (π-A) isotherm started after 10 min of lagging for complete chloroform evaporation. All the experiments were carried out at least three times. The physical states of the monolayers were estimated by the inverse of the compressibility modulus C_s_^−1^ that is obtained from the π-A isotherms calculated according to the following equation, where A is the mean area per molecule (Å^2^ · molecule^−1^), π the surface pressure (mN·m^−1^) and T the absolute temperature (K):

$$C_{s}^{-1}\;=\;-A{\left(\frac{d\pi }{dA}\right)}_{T}$$

### 3.8. Biocompatibility of the HA-ADH-Ag NPs

Human fibroblast cell line BJ5tα and human keratinocytes cell line HaCaT were used to determine the toxicity of the hybrid NPs towards mammalian cells as previously described. NPs suspensions were put in contact with the previously cultured in a 96-well plate (6 × 10^4^ cells per plate) cells, and after 24 h the particles were removed and the cells were let for 24 h to recover. Thereafter, the number of viable cells was detected using alamarBlue assay kit. All results are reported as mean value ± standard deviation (*n* = 3). In parallel, 20 µL of a PBS solution containing 0.1% (*v*/*v*) of calcein AM and 0.1% (*v*/*v*) of ethidium homodimer-1 were added to each well and the cells were stained for 15 min in the dark. The stained cells were observed using a fluorescence microscope (Nikon/Eclipse Ti-S, Amstelveen, The Netherlands), the stained live cells are displayed in green and the dead cells in red.

## 4. Conclusions

Silver NPs have been postulated as a powerful tool against the rising drug-resistant bacteria. In this work, we developed a safe to human cells antimicrobial nanocomposite through reduction of silver nitrate with HA-ADH of increasing Mw. The adipic acid-modified polymer was not only used as a silver reducing agent to obtain Ag NPs, but also stabilised the NP dispersion, decreased the NPs toxicity to human cells and enhanced their antimicrobial efficacy. TEM analysis revealed that the Mw of the biopolymer determined the size, morphology and polydispersity of the NPs, being the ones produced with medium molecular weight polymer (600 and 200 kDa) smaller and less polydisperse than those produced with 40 kDa or 2 MDa HA-ADH. The hybrid HA-ADH-Ag NPs showed strong antimicrobial effect towards the planktonic form of Gram-negative *E. coli* and Gram-positive *S. aureus* without affecting the viability of fibroblast and keratinocytes cells. Mw-related antimicrobial activity of the HA derivatives and developed nanocomposites was studied through Langmuir isotherms. Interestingly, higher Mw of HA-ADH in bulk form, presented greater interaction with a model bacterial membrane. The Langmuir characterisation of NPs-*E. coli* model membrane interaction also showed correlation with their antimicrobial activity. At physiological membrane pressure, 200 and 600 kDa HA-ADH-Ag NPs displayed the strongest interaction, followed by 2 MDa and 40 kDa. From low to medium Mw HA-ADH-generated NPs, the antibacterial capacity is clearly influenced by higher polymer Mw. On the contrary, although strong effect of 2 MDa HA-ADH in bulk form was observed in the Langmuir isotherm, the low interaction of the corresponding NPs, being larger and irregular in shape, with the model membrane confirmed the role of the NP size/morphology in their antimicrobial activity. Thus, the polymer Mw not only determines the NPs synthesis outcomes in terms of size, stability and dispersity, but also endowed them with the ability to interact with the bacterial membrane conferring enhanced antibacterial activity.

## Data Availability

All data generated or analysed during this study are included in this manuscript and its supplementary information files.

## Supplementary Materials

The following are available online at https://www.mdpi.com/article/10.3390/ijms222413428/s1.
