# Supplementary material for: Hyaluronic Acid Derivative Molecular Weight-Dependent Synthesis and Antimicrobial Effect of Hybrid Silver Nanoparticles
Source: Int J Mol Sci. 2021 Dec 14;22(24):13428. doi: 10.3390/ijms222413428 (PMC8707691; doi:10.3390/ijms222413428)
Supplement: Supplementary file 1 [file ijms-22-13428-s001.zip › ijms-1499648-supplementary-for publish.pdf]

International Journal of
*Molecular Sciences*

MDPI

*Supplementary Materials*

# Hyaluronic Acid Derivative Molecular Weight-Dependent Synthesis and Antimicrobial Effect of Hybrid Silver Nanoparticles

**Guillem Ferreres, Sílvia Pérez-Rafael, Juan Torrent-Burgués and Tzanko Tzanov ***

Grup de Biotecnologia Molecular i Industrial, Department of Chemical Engineering, Universitat Politècnica de Catalunya, Rambla Sant Nebridi 22, 08222 Terrassa, Spain; guillem.ferreres@upc.edu (G.F.); silvia.perez.rafael@upc.edu (S.P.-R.); juan.torrent@upc.edu (J.T.-B.)

* Correspondence: tzanko.tzanov@upc.edu

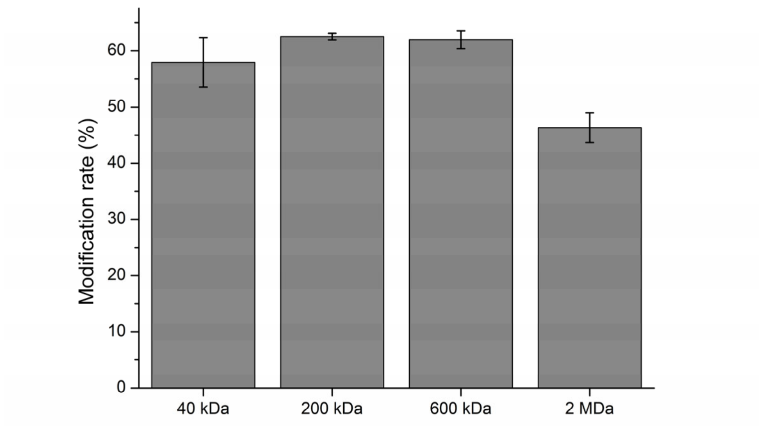


**Figure S1.** Modification degree of the HA-ADH evaluated by the TNBSA assay.

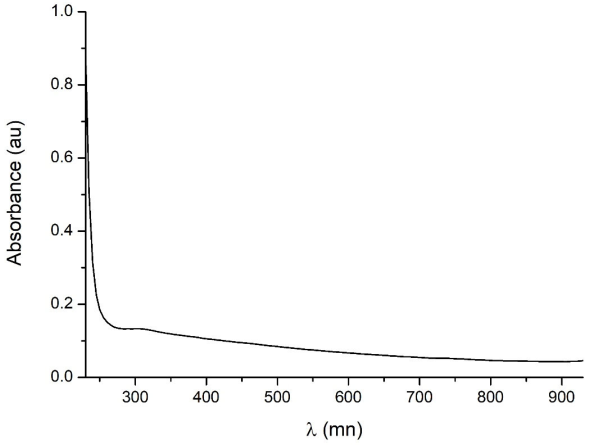


**Figure S2.** UV-vis spectrum of unmodified 200 kDa HA after 24 h of incubation with silver nitrate.

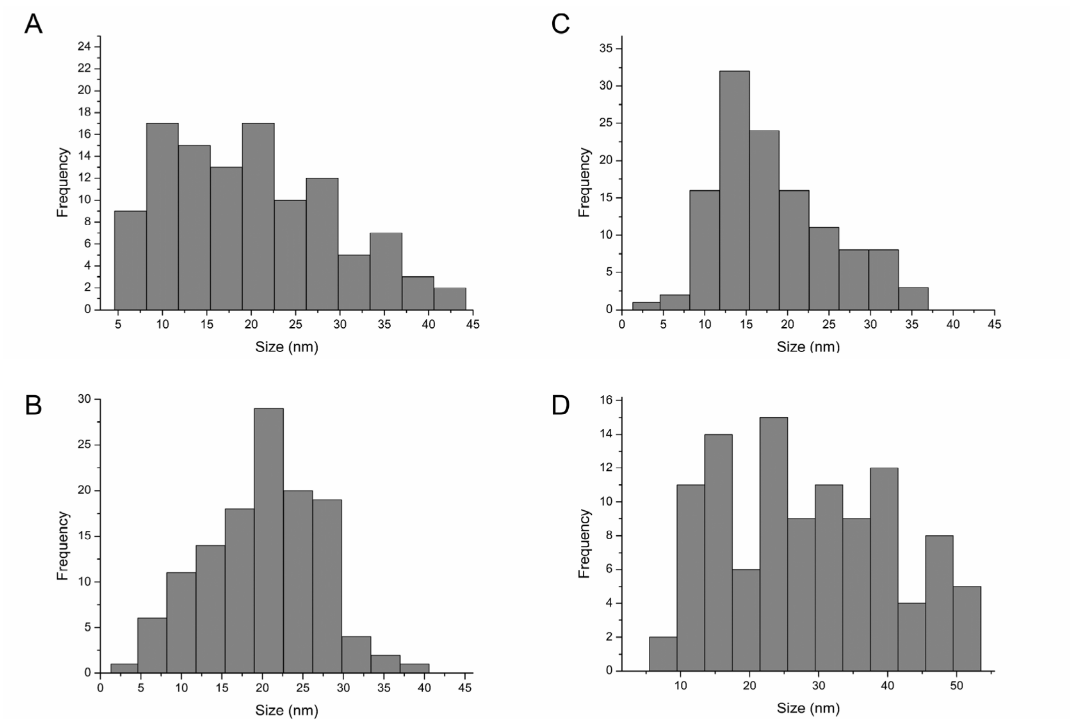


**Figure S3.** Size histogram of HA-ADH-Ag NPs synthesised with A) 40 kDa, B) 200 kDa, C) 600 kDa and D) 2 MDa.

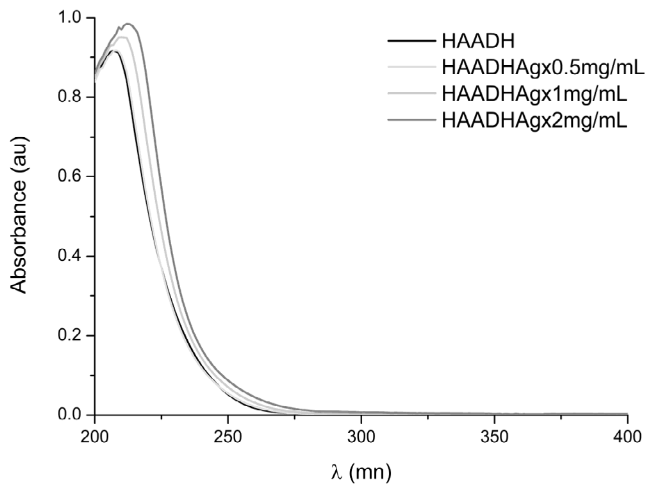


**Figure S4.** UV-vis spectra of HA-ADH with different concentrations of silver nitrate.

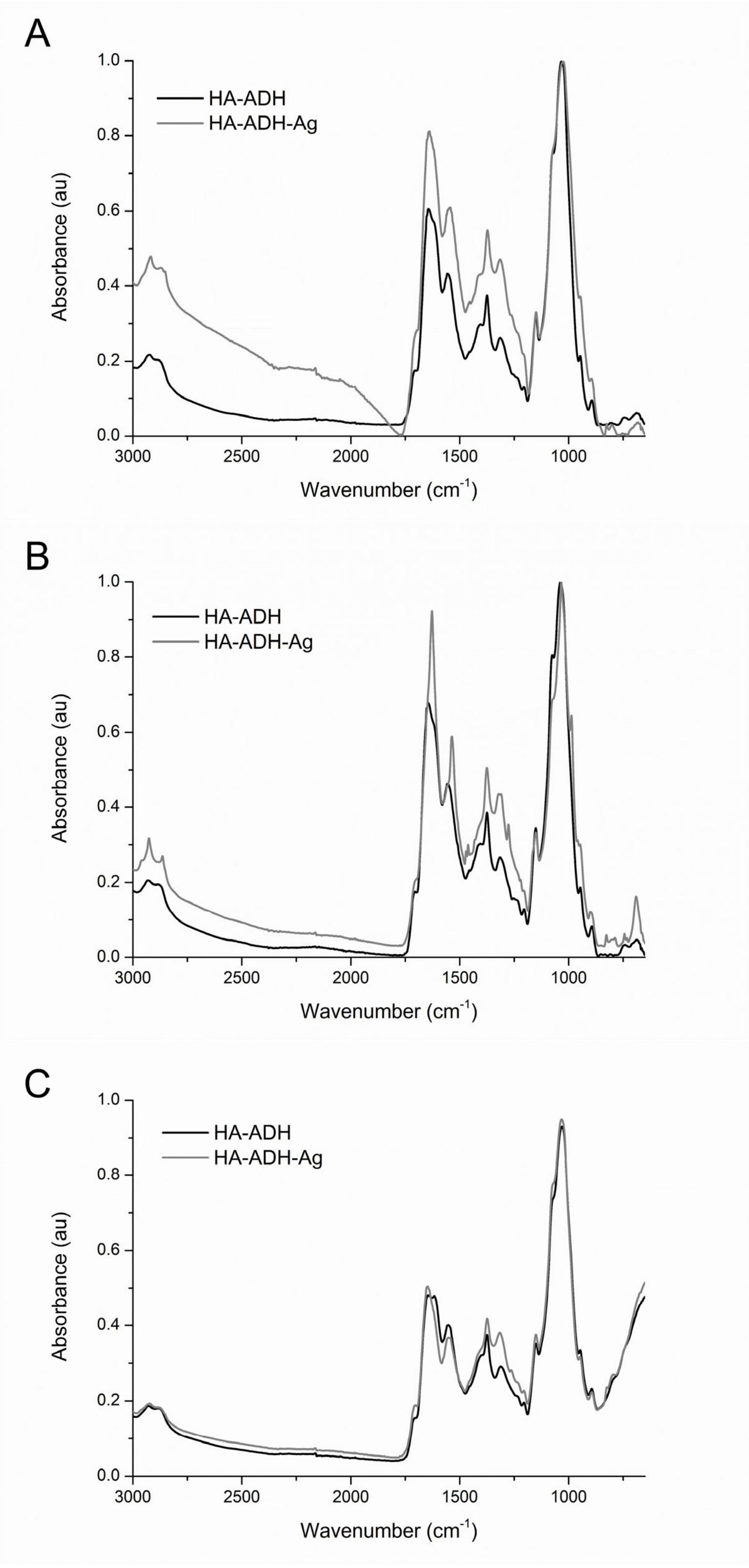


**Figure S5.** FTIR spectra of HA-ADH and HA-ADH-Ag NPs, A) 40 kDa HA-ADH , B) 600 kDa HA-ADH and C) 2 MDa HA-ADH.

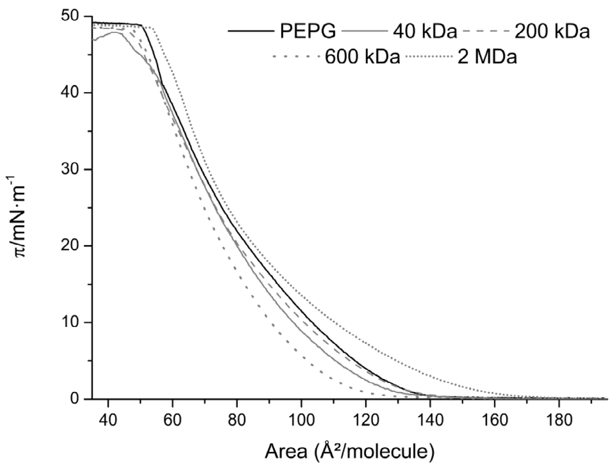


**Figure S6.** π-A isotherms of a PE-:PG 8:2 mix monolayers in PBS subphase (solid black), and with 40 kDa (grey solid), 200 kDa (grey dash), 600 kDa (grey dot) and 2 MDa (grey short dot) unmodified HA.

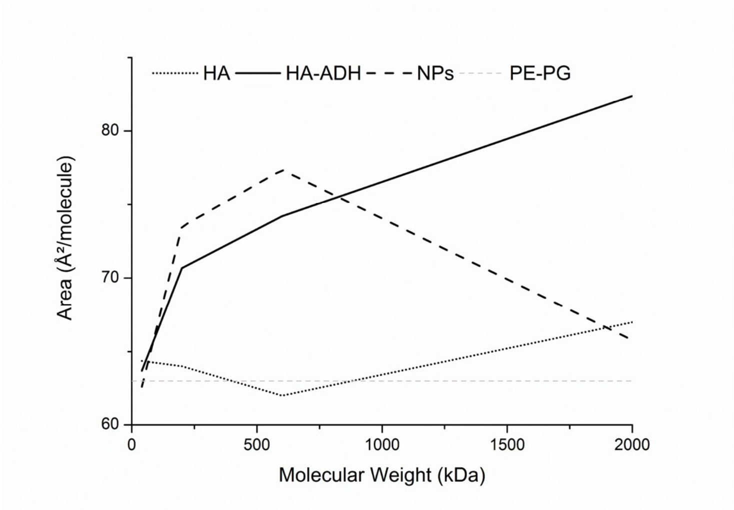


**Figure S7.** Representation of Area per molecule values at 33 mN·m$^{-1}$ vs Mw of unmodified HA, HA-ADH and HA-ADH-Ag NPs. Dash grey line represent the area of the control PE:PG isotherm at this pressure.

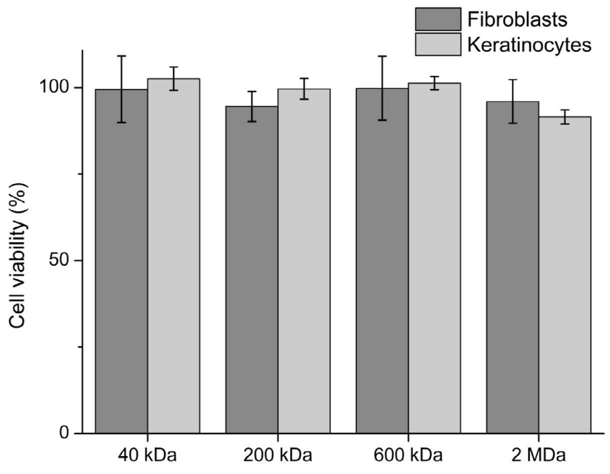


**Figure S8.** Cytotoxicity of HA-ADH in a concentration of 0.9 % (w/v) towards fibroblasts (BJ5tα) and keratinocytes (HaCat) assessed by alamarBlue assay.
